# PPAR*γ* and Its Agonists in Chronic Kidney Disease

**DOI:** 10.1155/2020/2917474

**Published:** 2020-02-25

**Authors:** Yuhua Ma, Manman Shi, Yuxin Wang, Jian Liu

**Affiliations:** ^1^Department of Nephrology, Traditional Chinese Medicine Hospital of Kunshan, Kunshan, Jiangsu, China; ^2^Department of Nephrology, Shanghai Ruijin Hospital, Shanghai Jiao Tong University School of Medicine, Shanghai, China

## Abstract

Chronic kidney disease (CKD) has become a global healthcare issue. CKD can progress to irreversible end-stage renal diseases (ESRD) or renal failure. The major risk factors for CKD include obesity, diabetes, and cardiovascular diseases. Understanding the key process involved in the disease development may lead to novel interventive strategies, which is currently lagging behind. Peroxisome proliferator-activated receptor *γ* (PPAR*γ*) is one of the ligand-activated transcription factor superfamily members and is globally expressed in human tissues. Its agonists such as thiazolidinediones (TZDs) have been applied as effective antidiabetic drugs as they control insulin sensitivity in multiple metabolic tissues. Besides, TZDs exert protective effects in multiple other CKD risk disease contexts. As PPAR*γ* is abundantly expressed in major kidney cells, its physiological roles in those cells have been studied in both cell and animal models. The function of PPAR*γ* in the kidney ranges from energy metabolism, cell proliferation to inflammatory suppression, although major renal side effects of existing agonists (including TZDs) have been reported, which limited their application in treating CKD. In the current review, we systemically assess the function of PPAR*γ* in CKDs and the benefits and current limitations of its agonists in the clinical applications.

## 1. Introduction

Chronic kidney disease (CKD) has become one of the most common diseases in the world. Chronic renal failure is the end stage of the progression of various types of CKD. In Europe and the United States, the number of patients with end-stage renal disease (ESRD) is rapidly increasing at a rate of 5%–8% per year [1]. ESRD is pathologically characterized by renal fibrosis, including glomerular sclerosis and renal interstitial fibrosis. Renal fibrosis is almost the result of all progressive kidney diseases and is a complex common chronic process involving multiple mechanisms such as extracellular matrix (ECM) and cytokine secretion, energy metabolism, podocyte injury, cell proliferation and differentiation, endoplasmic reticulum stress, autophagy, infiltration of inflammatory cells, and activation of fibroblasts [2].

## 2. PPAR*γ* Structure and Function

Peroxisome proliferator-activated receptors (PPARs) are ligand-activated transcription factors (TFs) of nuclear hormone receptor superfamily [3]. Three PPAR subtypes (PPAR*α*, PPAR *β*/*δ*, and PPAR*γ*) have been found in different species. In humans, the PPAR*γ* gene is located on chromosome 3 (3p25.2) and contains 9 exons expanding more than 100 kb, which generates 4 PPAR*γ* variants (*γ*1, *γ*2, *γ*3, and *γ*4) [4]. These isoforms are expressed differently in different tissues. PPAR family proteins share structural similarities, all containing a C-terminus ligand-binding domain which responds to ligand stimulation and controls heterodimerization with retinoid X receptors (RXR) upon activation [5]. Ligand stimulation leads to posttranslational modifications in PPAR*γ* through MAPK and PI3K pathways [6], and this is followed by conformational changes to a proactive form. The N-terminus DNA-binding domain recognizes and binds to specific responsive elements to modulate gene transcription [5]. Additional regulatory mechanisms involve coregulator complexes which recruit and release dynamically to control PPAR activation in the chromatin level [7]. For PPAR*γ*, it interacts with multiple published coregulators in energy metabolism, and these include coactivators of steroid receptor coactivator 2 (SRC-2), PPAR*γ* coactivator 1 (PGC-1) and receptor interacting protein 140 (RIP140), and PR domain containing 16 (PRDM16), as well as corepressors such as nuclear receptor corepressor 1 (NCOR1) and NCOR2 [8]. In addition, PPAR*γ* represses inflammatory TF activations, a process referred to as transrepression [9]. For example, the PPAR*γ* agonist inhibits NF-kB, activator protein-1- (AP-1-) mediated inflammatory responses in multiple tissues [10]. More TFs have been reported to be coregulated by PPAR*γ*, and these include the CCAAT/enhancer binding protein (C/EBP) family [11], SREBP [12], REV-ERBa [13], and GATA3 [14]. Due to its wide-spectrum of regulatory effects, mutations in PPAR*γ* have been identified in the humans and led to dysfunctional lipid and glucose metabolism, insulin resistance, which developed into obesity-induced T2DM, dyslipidemia, NAFLD, and cancer [15–18].

## 3. PPAR*γ* and Kidney Fibrosis

PPAR*γ* is abundantly expressed in the medullary collecting duct and paraurethral and bladder epithelial cells [19], and it is also expressed in podocytes, mesangial cells, and vascular endothelial cells [20]. Although not fully delineated, accumulating studies have suggested that PPAR*γ* plays an important role in regulating physiological functions of the kidney [21]. Current studies using PPAR*γ* null mice or agonists have been concluded in Table 1.

**Figure 1:**
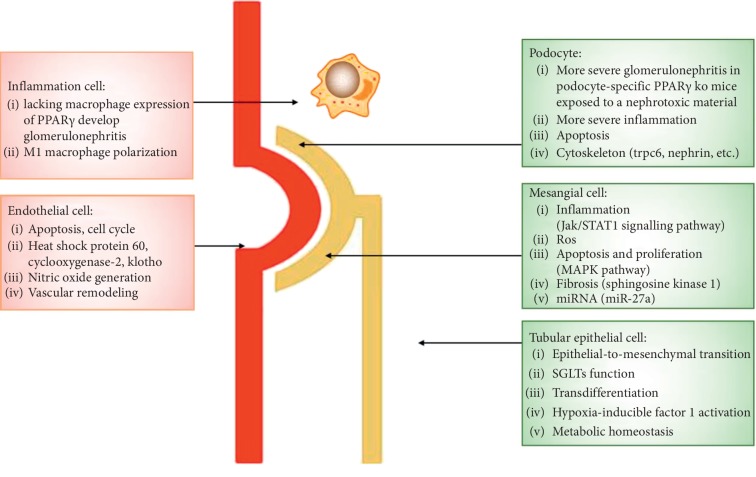
PPAR*γ* function in kidney.

The antifibrotic role of PPAR*γ* has been well accepted (Figure 1). Treatment of mice with synthetic PPAR*γ* ligands was shown to attenuate experimentally induced hepatic, cardiac, and kidney fibrosis [28]. The antifibrotic and renoprotective effects of the PPAR*γ* agonists pioglitazone and troglitazone were also confirmed in type 2 diabetes (T2D) rat models [29, 30] and unilateral ureteral obstruction (UUO) mice [31, 32] models, respectively.

Multiple mechanisms are potentially involved in the renoprotective effects of PPAR*γ* activation.

First, PPAR*γ* inhibits cell proliferation and apoptosis. It was reported that PPAR*γ* agonist pioglitazone inhibits mesangial cell proliferation and reduces proteinuria by downregulating *p*27, *Bcl-*2 (B-cell lymphoma 2) expression, and mitogen-activated protein kinase (MAPK) phosphorylation [33]. This was confirmed by a separate study using another PPAR*γ* agonist ciglitazone. The *in vitro* treatment of ciglitazone inhibited the proliferation of mouse mesangial cells induced by the platelet-derived growth factor (PDGF) by directly affecting the MAPK pathways linked with the downregulation of *p*21 and *cyclin D*1 and exerting the antiglomerular sclerosis effect [34]. Pretreatment with rosiglitazone significantly inhibited mesangial cell hypertrophy and proliferation in diabetic rats [35]. The effect of thiazolidinediones (TZDs) on puromycin aminonucleoside nephrosis (PAN) increased WT-1 expression, inhibited its necrosis and apoptosis, which may be related to up-regulation of *Bcl-xl* expression and inhibition of Caspase-3 activity, and decreased TGF-beta [36]. PPAR*γ* agonists can not only inhibit the apoptosis of endothelial cells induced by tumor necrosis factor (TNF) but also inhibit the spontaneous apoptosis of endothelial cells and reduce the damage of glomerular capillary endothelium [37].

Second, PPAR*γ* inhibits inflammation in the kidney diseases.

Inflammation is one of the major causes of the kidney injury and decline of kidney function. Inflammatory markers, such as C-reactive protein and inflammatory cytokines, correlate with the end stage of kidney disease [38]. Activation of PPAR*γ* alleviates inflammation in different types of kidney cells. A synthesized PPAR*γ* ligand 15d-PGJ2 was reported to inhibit the chemokines induced by interferon-7 (INF-7) by inhibiting the JAK/STAT1 signaling pathway in mesangial cells [39]. Rosiglitazone activates PPAR*γ* SUMOylation, inhibiting NCoR degradation and NF-*κ*B activation in LPS-stimulated renal proximal tubular cells, which in turn decreases IL-8 and MCP1 expression [40]. PPAR*γ* agonist (rosiglitazone, troglitazone, and thiazolidinedione) attenuates excessive inflammatory response in activated proximal tubular epithelial cells in IgAN through suppressing ATR1 expression [41]. Loss of PPAR*γ* abundance and transcriptional activity was observed in glomerular podocytes in experimental RPGN. Blunted expression of PPAR*γ* in podocyte nuclei was also found in kidneys from patients diagnosed with crescentic GN [42].

Third, PPAR*γ* represses transforming growth factor-*β* (TGF-*β*) pathways. TGF-*β* signaling pathways play an important role in kidney fibrosis. Studies have shown that rosiglitazone effectively blocked fibrotic responses elicited by TGF-*β* in explanted fibroblasts and attenuated bleomycin-induced skin fibrosis *in vivo* [43]. Subsequent studies in a variety of cell types and model systems have confirmed and expanded these findings [44]. PPAR*γ* activation inhibits plasminogen activating-inhibiting factor-1 (PAI-1) and TGF-*β* in human mesangial cells [45]. The expression of the extracellular matrix is reduced, and the mechanism may be partly through the positive regulation of PPAR*γ* on hepatocyte growth factor (HGF), causing increased endogenous HGF, thereby inhibiting TGF- *β*/SMAD pathways [46]. PPAR*γ* agonists (15d-PGJ2, troglitazone, and ciglitazone) can inhibit TGF-*β*/SMAD signaling pathways to alleviate renal fibroblast activation, resulting in decreased expression of connective tissue growth factor (CTGF) and extracellular matrix synthesis [47], all contributing to reduced renal fibrosis progression.

Besides, PPAR*γ* can be influenced by epigenetic modification in kidney diseases. MicroRNA-27a induces mesangial cell and podocyte injury by PPAR*γ* in diabetic nephropathy (DN). And, micro-RNA23 can be mediated by PPAR*γ* in renal calcium oxalate crystal formation. lncRNA TUG1 could modulate ECM accumulation in DN by regulating miR-377 targeting PPAR*γ* [48–50]. However, the role of the epigenetic modification in PPAR*γ* function in kidney diseases still needs further exploration.

## 4. PPAR*γ* in Renal Glucose Control

PPARγ is important for regulating systemic glucose homeostasis. PPAR*γ* activation improves peripheral insulin sensitivity partly by increasing beneficial adipokines [51] (i.e., adiponectin, resistin, and leptin) and myokines [52] and reducing inflammatory cytokines (such as TNF-*α*) in adipose tissue and muscles [53]. It also induces the expression of key genes involved in glucose-stimulated insulin secretion in pancreatic beta cells [54]. Besides, glucose filtration, reabsorption, and gluconeogenesis in the kidney regulated by PPAR*γ* are essential for glucose metabolism. A recent study showed that PPAR*γ* agonist rosiglitazone induced the hypoglycemic effects through inhibition of gluconeogenesis on proximal tubule cells [55]. The transport of glucose in kidney depends on sodium-dependent glucose cotransporters (SGLTs), localized in the epithelial cells of the proximal renal tubules, and glucose transporters (GLUTs), localized in the basolateral membrane [56]. SGLT2 is a well-characterized cotransporter in SGLTs family and primarily found in renal tissues. About 90% of glucose filtered from glomeruli is absorbed by SGLT2, and mutations of this gene are associated with elevated urine glucose, suggesting that SGLT2 is the key factor for glucose reabsorption in the kidney [57]. SGLT2 can inhibit increased secretion of glucagon from pancreatic *α* cells induced in hyperglycemia, which in turn is inhibited by high glucose environment [58]. Troglitazone, as a PPAR*γ* agonist, can increase the expression of SGTL2 in the renal proximal tubules and reverse the downregulation of SGLT2 expression and upregulation of glucagon secretion induced by high glucose [59, 60]. Accordingly, PPAR*γ* antagonist (GW9662) decreased the expression of SGLT2 and increased glucagon, similar with high glucose [59]. Those effects were potentially mediated through PI3K/Akt signaling pathways, as hyperglycemia increased both PI3K and Akt phosphorylations (P-AKT) in alpha cells which contributed to the SGLT2 expression [59]. Troglitazone treatment decreased PI3K and P-AKT in high glucose-treated alpha cells. In addition, PI3K inhibitor reversed high glucose-induced SGLT2 decrease and glucagon increase in alpha TC cells [59].

When glucose has been transported to the renal tubular epithelial cells and the concentration is higher than that in the renal interstitial, it diffuses to the renal interstitial through the GLUTs on the basolateral membrane. Studies have suggested that mice with GLUT2 deletion present with glycosuria and human with GLUT2 mutations present with Fanconi syndrome, glycosuria, and proximal tubule dysfunction [61].

## 5. PPAR*γ* in Renal Lipid Metabolism

An important biological function of PPAR*γ* is its involvement in lipid metabolism. PPAR*γ* can be activated by fatty acids and exogenous peroxisome proliferators to regulate the expression of enzymes conducting lipid metabolism [62]. Increased expression of PPAR*γ* was found in multiple tissues in diet-induced obesity models (DIO) [63]. PPAR*γ* can be induced before transcription activation of adipocyte genes and plays an important role in adipocyte differentiation [64]. PPAR*γ* localizes at the brown-fat-specific enhancers, and the binding of nuclear factor I-A (NFIA) to the brown fat enhancers precedes and facilitates the binding of PPAR*γ*, leading to increased chromatin accessibility and active transcription [65]. PPAR*γ* can also regulate the mutual transformation between adipocytes and osteoblasts, thereby affecting lipid metabolism, which can promote the differentiation of bone marrow mesenchymal stem cells into adipocytes and inhibit the differentiation into osteoblasts [66]. Several studies which reported mutations in the PPAR*γ* protein were related to some diseased states; for example, PPAR*γ* P115Q results in severe obesity, and P467L and V290M mutations are partially involved in familial lipodystrophy type 3 [67].

The kidney is one of the target organs of PPAR*γ*-regulated lipid metabolism. All PPARs have been detected in rodent and human kidney, including glomeruli, medullary collecting duct, and pelvic urothelium. Renal proximal tubule expresses lipid metabolism-related enzymes that can upregulate transcriptional activity by sufficient expression of PPAR*γ* [68]. Kidney can process free fatty acids (FFAs), including reabsorption by proximal tubule and metabolism within mitochondria of proximal tubule [69]. FFAs are usually metabolized with the method of *β*-oxidation and then constitute the largest energy pool in the human body. Moorhead et al. were the first to report the relationship between lipid metabolism and kidney disease [70]. Mutations in lipid metabolism enzymes resulted in lipotoxicity and renal function deterioration [71]. Studies about animal models showed that PPAR*γ*-deficient mice presented lipid metabolic disorders. One earlier study using PPAR*γ*2 and leptin double knockout mice (POKO) showed that those mice presented spontaneous metabolic syndrome, hypertension, albuminuria, and renal dysfunction [72, 73]. Another study showed that lipotoxicity accelerates lipid accumulation and inflammation and then leads to glomerular diseases, which suggested that renal lipid metabolism may serve as a target for specific therapies aimed at slowing the progression of podocyte failure during metabolic syndrome [74].

## 6. PPAR*γ* Activation in Kidney Immune Cells

**Figure 2:**
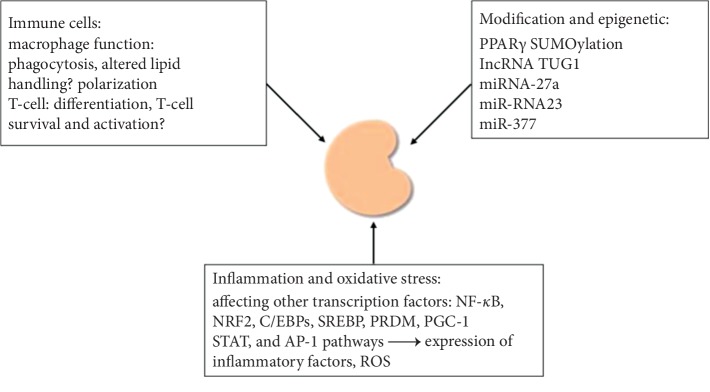
Indirect renal-protective effects.

Renal resident macrophages play important roles in kidney homeostasis and pathology. Macrophages are involved in the acute kidney disease and chronic kidney disease by adopting different type of phenotypes including M1 macrophage, subtypes of M2 macrophage, and MDSCs. While M1 cell promotes kidney injury, M2 cells contribute to the repairing response to the kidney injury (Figure 2). In kidney, the deletion of PPAR*γ* in hematopoietic cells enhances inflammatory renal disease in the anti-GBM antibody-induced glomerulonephritis mouse model [24]. Mice lacking macrophage expression of PPAR*γ* develop glomerulonephritis similar to autoimmune glomerulonephritis [75]. In addition, PPAR*γ* agonist pioglitazone decreased renal calcium oxalate crystal formation by suppressing M1 macrophage polarization [48], and it also promotes the differentiation of monocytes into M2-type macrophages [76]. Deletion of PPAR*γ* in the macrophages leads to impaired phagocytosis and macrophage polarization and altered lipid handling [77]. PPAR*γ* agonists downregulate the expression of inflammatory factors in monocytes and macrophages by inhibiting NF-kappaB (NF-*κ*B), signal transducer and activator of transcription (STATs), and AP-1 pathways. PPAR*γ* agonists (15d-PGJ2) block the proinflammatory effects of IFN-*γ* by inhibiting the JAK/STAT (Janus kinase/signal transducer and activator of tranions) pathways [78]. In the rat model of type 2 diabetes, pioglitazone significantly reduces macrophage infiltration in renal tissue along with inflammatory and fibrotic factors such as NF-*κ*B, chemokine (C-C motif) ligand 2 (CCL2) TGF-B, PAI, and vascular endothelial growth factor (VEGF) [29].

In addition, PPAR*γ* activation also modulates the function of other types of kidney-resident immune cells such as T cells [79]. Deletion of PPAR*γ* in Th17 cell caused loss of Th17 cell differentiation and predisposes to autoimmune diseases [80].

## 7. Hypertension and PPAR*γ*

Hypertension is both important cause and consequence of kidney diseases. Kidney function is always associated with increasing blood pressure (BP). In CRIC study, the prevalence of hypertension was 86%, much higher than the general population [81]. Hypertension was one of the independent risk factors for the progression of CKD.

Besides the wide distribution in adipocytes, adrenal gland, spleen, muscle, etc., PPAR*γ* is also widely expressed in vascular endothelial cells and smooth muscle cells and regulates smooth muscle proliferation, migration and apoptosis, inflammation, atherosclerosis, and other pathologic processes [82]. Studies both *in vivo and in vitro* supported that PPAR*γ* plays a vital protective role in cardiovascular disorders. In the study of DOCA-salt-hypertensive rats, rosiglitazone was effective in preventing the increased blood pressure and partially improved endothelial dysfunction [83]. Recent studies observed increased expression of PPAR*γ* in blood vessels of spontaneously hypertensive rats, which is beneficial to the vascular function and lowers the blood pressure [84]. PPAR*γ* can also inhibit the growth of vascular cells, induce apoptosis of vascular smooth muscle cells (VSMC), and improve the vascular structure [85]. Nicol et al. has shown the ability of lowering blood pressure (BP) by endothelial-specific PPAR*γ* activation in the context of a high-fat diet mouse model [86]. Benkirane et al. figured out that PPAR*γ* might be responsible for the amelioration of vascular remodeling in hypertension, the mechanisms of which may include inhibiting the expression of matrix metalloprotein-9 (MMP-9) and disturbing PI3K and MAPK signaling pathways [87, 88]. Recent studies reported that PPAR*γ* can regulate BP through renin-angiotensin-aldosterone system (RAAS), which acts as a negative regulator of Ang II receptor 1 transcription via the following pathways: inhibiting the expression of angiotensinogen, inhibiting Ang II activity, and degrading the angiotensin receptor I expression in the VSMCs [21, 82]. Clinical researchers have found a significant reduction in blood pressure after the rosiglitazone treatment in type 2 diabetic patients [89]. The data by Barroso et al. figured out that PPAR*γ*-dominant negative mutations are associated with hypertension [90]. In addition, PPAR*γ* is also important to circadian variations of blood pressure and heart rate through *Bmal1*. It controls cardiovascular rhythms via an influence on sympathetic nerve activity and the molecular clocks. PPAR*γ* also protects against age-related hypertension [91].

Based on those functions, activators of PPARs (TZDs) may become therapeutic agents useful for the prevention of cardiovascular disease beyond their effects on carbohydrate and lipid metabolism. Although concerns of the side effects, such as the systemic fluid retention, have been proposed, *Scnn*1*g*, identified as a critical PPAR*γ* target gene, has played an important role in the control of edema [23, 92].

## 8. Clinical Application of PPAR*γ* Agonist in Kidney Diseases

PPAR*γ* agonists have shown great clinical effects in treating metabolic disorders. Many agonists have been developed, including 15-deoxy-Δ12, 14-prostaglandin J2 (15dPGJ2), and TZDs [93]. PPAR*γ* agonists appear to be useful in reversing this early stage of the renal fibrosis (epithelial-mesenchymal transition, EMT) in the condition of the high glucose and to restore the function of SGLT-proteins mediated glucose uptake [56, 60]. TZDs, which used in the treatment of T2D, were proved to indirectly slow down the progression of renal disease by improving glucose intolerance and reducing the urinary albumin [21, 94]. A meta-analysis involving 2860 diabetic patients demonstrated that TZDs produced significant decreases in the levels of urinary albumin, which was in addition to that of RAS blockade because practically all patients in the latter studies were treated with ACEI or ARBs [95].

Accumulating evidence suggested that PPAR*γ* agonists could also provide a protection in wider spectrum of kidney diseases, such as the acute nephrotic syndrome, nondiabetic glomerulosclerosis, and the polycystic kidney [96, 97]. Pioglitazone was reported to improve the renal functions in animal models of renal ischemia reperfusion-induced AKI via regulating *Tnf* and *Nrf*2 expression [98]. Furthermore, recent studies have highlighted beneficial roles of PPAR*γ* agonists for patients with chronic renal failure, hemodialysis, as well as peritoneal dialysis (PD) could also benefit from [99, 100]. Liu et al. pointed out that rosiglitazone could protect against high phosphate-induced vascular calcification in CKD mice [101]. Zhang et al. provided the evidence that rosiglitazone had the protective effects on rat peritoneal mesothelial cells against PD solution-induced damage probably by inhibiting inflammation and regulating AQP 1 and ZO 1 gene expressions [102].

Interestingly, as a member of the RAAS inhibitors is widely used in the treatment of renal disease, telmisartan (characterized as selective PPAR*γ* modulators in 2005) had advantages in reducing albuminuria, serum creatinine, and glomerulosclerosis in a nondiabetic model by its partial agonistic activity on PPAR*γ*. Besides, treatment with telmisartan confirmed the protective effects against daunorubicin- (DNR-) induced nephrotoxicity by reducing the level of Ang II and ET-1 expression, which was associated with reduced inflammation and oxidative stress in part through the activation of PPAR*γ* [103–105].

Previous studies have shown that PPAR*γ* agonists may have some protective effects on CVD end of CKD. In a PROactive study, CKD patients treated with pioglitazone were less likely to reach a composite end point of all-cause death, MI, and stroke [106]. These effects may be related to anti-inflammation effect and protection of endothelial cells. Short-term rosiglitazone therapy reduced insulin resistance, markers of inflammation, and abnormal endothelial function in patients with CKD [107]. In nondiabetic ESRD patients, pioglitazone significantly changes the visceral-subcutaneous fat distribution and improves the adipokine profile with a decrease in hepatic insulin resistance [108]. In nondiabetic renal allograft recipients, pioglitazone treatment reduces the progression of carotid IMT and improves insulin resistance [109]. However, the protective effect of PPAR*γ* agonists on the progress of renal function needs further exploration.

## 9. Side Effects of PPAR*γ* Agonists in Kidney

Despite the evidence that PPAR*γ* agonists could obviously bring benefits to renal functions as stated above, these beneficial effects were shadowed by the risk for fluid retention, peripheral edema, or blood volume expansion [92], which could increase various degrees of burdens on kidney. For example, some studies proved that TZDs contributed the fluid retention by altered sodium and water reabsorption in the distal collecting ducts of the kidney [23, 110]. Clinical research figured out that uncomplicated diabetic patients' combined use of RAAS inhibitors and PPAR*γ* agonists promotes anemia [111]. Besides, people with long-term TZDs drug treatment are also peculiarly prone to the osteoporosis and heart failure [112]. Safety warnings and even drug withdrawal in Europe have come out because of the increasing risk in bladder cancer by pioglitazone [113–115]. A combined therapy including PPAR*γ* agonists and other renoprotective measures appears to be more reasonable than one single intervention. Ongoing efforts have been made to identify more selective modulators of PPAR*γ* that reduce or eliminate the adverse effects of PPAR*γ* agonists.

## 10. Conclusion

Overall, PPAR*γ* is one of the ligand-activated transcription factor superfamily members and is abundantly expressed many types of kidney cells. It is involved in renal disease by participating in cell proliferation and apoptosis, TGF-*β* pathway, inflammation and oxidative stress, lipid metabolism, hypertension, and so on. Activation of PPAR*γ* improves kidney injury. The application as clinical treatment was restricted by the side effect. And, the cell-specific function of PPAR*γ* agonist was still unclear. So, understanding the mechanism of PPAR*γ* function, exploring the cell specific reaction to the activation of PPAR*γ* agonist, and developing a new type of PPAR*γ* agonist with less side-effect may help application of the PPAR*γ* agonist into clinical treatment and ameliorate the renal injury.

## Figures and Tables

**Table 1:** Cell type of PPAR gamma deletion and the phenotype.

Studies	Cell type of PPAR gamma deletion	Animal model	Phenotype
Desch et al. [22].	Juxtaglomerular (JG) cells of the kidney	RC-PPARgamma(fl/fl) mice	Reduced PPARgamma expression, stronger renin signal and higher renin mRNA levels and plasma renin concentration in RC-PPARgamma (fl/fl) mice than in littermate control RC-PPARgamma (wt/wt) mice

Zhang et al. [23].	Renal collecting duct	Collecting duct-specific deletion of PPARgamma mice	Mice with CD knockout of PPARG were resistant to the rosiglitazone- (RGZ-) induced increases in body weight and had diminished plasma volume expansion, PPARgamma expression and reduced urinary sodium excretion in response to RGZ

Chafin et al. [24].	Hematopoietic cell	Antiglomerular basement membrane (anti-GBM) mouse model	PPAR*γ* (–/–) mice had a more severe glomerular and tubulointerstitial disease, decreased CD4(+) CD25(+) regulatory T cells, increased CD8(+) : CD4(+) ratio and plasma interleukin-6 levels compared with the PPAR*γ*(+/+) animals

Toffoli et al. [25].	Epiblast-specific deletion	PPAR*γ* null mice	Renal hypertrophy, glucosuria, albuminuria, renal insufficiency with decreased creatinine clearance progress, renal fibrosis, mesangial expansion, and antiphospholipid syndrome

Yang et al. [26].	Systemic PPAR*γ* deletion	MoxCre/flox mice	Suppressed circadian variations in oxygen consumption, CO2 production, food and water intake, locomotor activity, and cardiovascular parameters

Zhou et al. [27].	Systemic PPAR*γ* deletion	Whole-body PPAR*γ* knockout mice	The null mice developed severe polydipsia and polyuria, reduced urine osmolality, modest hyperphagia, and progressive weight loss; after 24 h of water deprivation, the null mice had a lower urine osmolality, a higher urine volume, a greater weight loss, and a greater rise in hematocrit; the response of urine osmolality to acute and chronic 1-desamino-8-D-arginine vasopressin treatment was attenuated
